# Efficacy of *Ginkgo biloba* as an adjunct to donepezil in amyloid PET-positive Alzheimer’s patients

**DOI:** 10.3389/fneur.2025.1563056

**Published:** 2025-03-11

**Authors:** YoungSoon Yang, Min-Seong Koo, Yong Tae Kwak

**Affiliations:** ^1^Department of Neurology, Soonchunhyang University College of Medicine, Cheonan Hospital, Cheonan, Republic of Korea; ^2^Department of Psychiatry, Catholic Kwandong University College of Medicine, Incheon, Republic of Korea; ^3^Department of Neurology, Hyoja Geriatric Hospital, Yongin, Republic of Korea

**Keywords:** Alzheimer’s disease, amyloid PET, donepezil, *Ginkgo biloba*, MDS-Oaβ

## Abstract

**Background:**

*Ginkgo biloba* is widely used in some regions as an adjunct therapy for Alzheimer’s disease (AD). Its potential mechanisms include antioxidative and anti-amyloid properties, yet clinical evidence remains mixed.

**Objective:**

We investigated whether combining Ginkgo with donepezil confers additional benefits in amyloid PET-positive AD patients. We also explored changes in the plasma biomarker MDS-Oaβ (Multimer Detection System–Oligomeric Aβ), which reflects the propensity of Aβ monomers to form oligomers.

**Methods:**

This retrospective study included newly diagnosed, drug-naïve AD patients who were amyloid PET-positive and had at least 12 months of follow-up. Participants received either donepezil alone (Donepezil-only) or donepezil plus Ginkgo (Donepezil-Ginkgo). Clinical measures included the Korean version of the Mini-Mental State Examination (K-MMSE) and the Sum of Boxes of the Clinical Dementia Rating (CDR-SB). Plasma MDS-Oaβ was assessed at baseline and at 12 months.

**Results:**

A total of 101 patients were analyzed (60 Donepezil-only, 41 Donepezil-Ginkgo). Baseline demographics and clinical characteristics were similar. After 12 months, the Donepezil-only group showed minimal change in K-MMSE and a slight decrease in MDS-Oaβ. The Donepezil-Ginkgo group demonstrated a significant improvement in K-MMSE (+2.4) and a larger reduction in MDS-Oaβ (−0.15). No significant between-group difference was observed for CDR-SB. Adverse events were mostly mild and did not lead to discontinuation.

**Conclusion:**

The addition of Ginkgo to donepezil may yield superior cognitive outcomes and a greater decrease in plasma MDS-Oaβ compared with donepezil alone in amyloid PET-positive AD patients. Further large-scale, prospective trials are warranted to validate these findings and elucidate Ginkgo’s mechanistic role in AD.

## 1 Introduction

With the global increase in the aging population, the prevalence of Alzheimer’s disease (AD) is also rising. Despite the approval of memantine by the U.S. FDA in October 2003 and numerous research efforts over the past two decades, an effective and cost-efficient medications for AD remains elusive. Consequently, the re-evaluation of existing drugs already used in certain regions for Alzheimer’s disease or related dementia is emerging as another key axis in the development of new treatments.

*Ginkgo biloba* (Ginkgo; Ginexin-F^®^) is used in South Korea as an adjunct therapy to help prevent or treat dementia. In this study, we selected Ginexin-F^®^, a standardized *Ginkgo biloba* extract that has been approved by the Ministry of Food and Drug Safety (Approval No. MFDS-199702183, Available from: https://nedrug.mfds.go.kr/CEAAA21) for its stringent quality control. Ginexin-F® is standardized to contain 24% flavonoid glycosides and 6% terpene lactones, with ginkgolic acids maintained below 5 ppm, ensuring its compositional equivalence to EGb 761, which has been widely validated in numerous clinical studies. As Ginexin and EGb 761 share similar standardized compositions, findings from studies on EGb 761 are considered relevant. Ginkgo has shown various pharmacological effects, such as inhibiting oxidative stress, improving cerebral blood flow, reducing Aβ toxicity, and modulating neurotransmission and neuroplasticity (1). Despite these potential benefits, questions remain about whether Ginkgo truly confers clinical advantages for AD and, if so, by what mechanisms. While several preclinical studies suggest that *Ginkgo biloba* may act through antioxidative, anti-inflammatory, and anti-amyloid aggregation pathways (2, 3), the exact molecular basis remains unclear. Therefore, in our clinical study, we employed the plasma MDS-Oaβ biomarker as an indirect measure of amyloid oligomerization, which may partly reflect these underlying mechanisms.

Numerous studies have examined the role of *Ginkgo biloba* in preventing dementia and treating MCI and AD, yet the findings remain inconsistent. For example, a placebo-controlled pilot study demonstrated significant cognitive benefits when patient compliance was considered (4), whereas the GEM trial using EGb 761 in cognitively healthy elderly found no significant benefit—likely due to low dementia incidence and poor adherence (5). Similarly, the GUIDAGE trial in individuals with subjective memory impairment yielded inconclusive results (6, 7). Early meta-analyses indicated modest benefits (8), but a 2007 Cochrane review cast doubt on significant cognitive improvements (9). In contrast, more recent randomized trials and meta-analyses suggest that EGb 761 can improve cognition, neuropsychiatric symptoms, and activities of daily living in mild-to-moderate dementia (10), with a 12-month placebo-controlled study in China showing a significant reduction in dementia incidence among patients with amnestic MCI (11) and other studies reporting symptomatic improvements in MCI populations (12, 13). Nevertheless, overall results remain mixed, and a recent review concluded that definitive evidence of efficacy is still lacking (14). Possible explanations for these mixed outcomes include: misclassification of patients with non-AD dementia, leading to false positives in clinical trials and the difficulty in detecting small or slowly emerging drug effects within relatively short study periods. Conducting large, well-designed trials focusing on clinical outcomes alone is challenging, particularly given AD’s slow progression.

To address these issues, we designed a study comparing patients receiving donepezil monotherapy versus patients receiving donepezil plus Ginkgo, with two distinctive features: (1) inclusion of only amyloid PET-positive patients to increase diagnostic specificity for AD, and (2) use of the biomarker MDS-Oaβ (Multimer Detection System-Oligomeric Aβ) alongside clinical measures. MDS-Oaβ reflects the oligomerization propensity of Aβ in plasma, potentially capturing treatment effects at the pathological level. Hence, we aimed to determine whether adding Ginkgo to donepezil offers clinical or biomarker-based benefits in AD.

## 2 Materials and methods

### 2.1 Participants and methods

The Dementia Clinic at Soonchunhyang University College of Medicine has maintained the Soonchunhyang Dementia Registry for all dementia patients since March 2020. This registry includes diagnostic evaluations at the time of patient visits: a complete medical history, physical and neurological examinations, comprehensive neuropsychological testing, and routine laboratory tests including ApoE. Magnetic resonance imaging (MRI) is performed within 3 months, and 18F-FC119S PET/computed tomography (CT) is performed when possible in the same period.

From this registry, we identified patients who (1) visited between February 2020 and May 2024, (2) were newly diagnosed with probable AD per the NINCDS-ADRDA criteria (15), (3) tested positive on 18F-FC119S PET (amyloid PET positive), and (4) had at least 12 months of follow-up. All included patients were drug-naive for AD treatments such as cholinesterase inhibitors. After diagnosis, they received either donepezil alone (Donepezil-only group) or a combination of donepezil and Ginkgo (Donepezil-Ginkgo group).

We utilized Ginexin, a standardized *Ginkgo biloba* extract containing 24% flavonoid glycosides and 6% terpene lactones. For convenience, we refer to it as “Ginkgo.” Ginkgo was administered at a fixed daily dose of 240 mg, and donepezil started at 5 mg/day. The neurologist could raise the donepezil dose to 10 mg/day between 3 and 6 months if tolerated. In this naturalistic study, the clinician determined the dose adjustment method based on their judgment of potential tolerability and efficacy according to the patient’s characteristics. The Korean version of the Mini-Mental State Examination (K-MMSE), the Sum of the Boxes of the Clinical Dementia Rating (CDR-SB) and plasma MDS-Oaβ levels were the primary outcome measures. K-MMSE, CDR-SB, and plasma MDS-Oaβ levels, assessed at baseline and again after approximately 12 months. Because this was a retrospective, naturalistic study, slight variations in follow-up intervals were possible.

This study was based on retrospective registry data. Ethical approval was obtained from the Institutional Review Board of Soonchunhyang University Cheonan Hospital (IRB No. 2023–02–038-002), and informed consent was waived due to anonymized data use. The study was conducted in accordance with the Declaration of Helsinki.

### 2.2 Safety assessments

Adverse events were elicited at each visit by inquiring about the patient’s and caregiver’s observations, and by direct clinical observation. All adverse events reported or observed were documented.

### 2.3 The measurement of oligomerization of Aβ in plasma

Oligomerization of Aβ in plasma was assessed using the MDS-Oaβ method (16, 17). This test, like other assessments, was conducted at the time of initial enrollment according to the registry protocol and was followed up after 12 months. Prior to the assay, plasma samples were initially thawed at a temperature of 37°C for a duration of 15 min. After this, synthetic amyloid-beta (Aβ made by PeopleBio Inc.) was added to the samples, followed by an incubation period of 48 h at 37°C. The incubated plasma sample mixture and serially diluted standard samples were added to their respective wells, and the plates were incubated at room temperature for 1 h. Subsequently, 100 μL/well of an enhanced chemiluminescence substrate solution (Rockland Immunochemicals Inc., Limerick, PA, United States) was added, and the Relative Luminescence Unit (RLU) values were determined using a Victor 3 spectrophotometer to quantify oligomerized Aβ.

### 2.4 Statistical analysis

Between-group differences (Donepezil-only vs. Donepezil-Ginkgo) were examined using chi-square tests for categorical variables and *t*-tests for continuous variables. Baseline demographic and clinical variables were compared, and changes in primary outcomes over 12 months were evaluated using independent *t*-tests. Within-group changes were analyzed using paired *t*-tests. Statistical analyses were performed using SPSS version 24.0 (SPSS Inc., Chicago, IL, United States).

## 3 Results

### 3.1 Baseline demographics and clinical characteristics of study subjects

**Figure 1:**
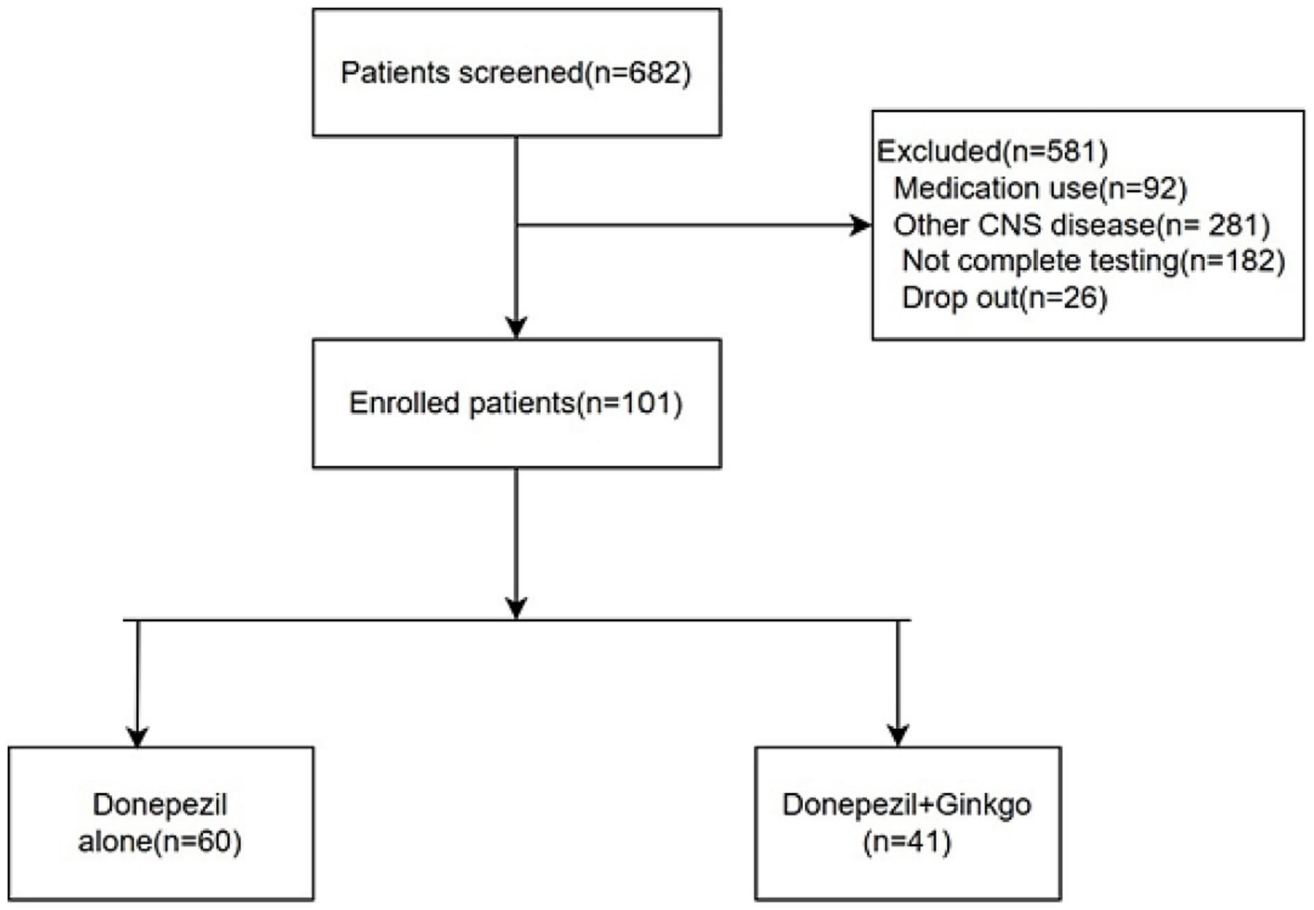
Flow chart of patients eligible for the study.

Among the 101 patients included in the analysis, 60 were treated with donepezil monotherapy, and 41 were treated with a donepezil plus Ginkgo (Figure 1). Although the Donepezil-Ginkgo group tended to have lower baseline K-MMSE scores (21.2 vs. 22.7), the difference was not statistically significant (*p* = 0.196). No other demographic or clinical differences were observed between the two groups. Baseline MDS-Oaβ values were 0.88 in the Donepezil-only group and 0.87 in the Donepezil-Ginkgo group, both exceeding the cutoff value of 0.78 for AD diagnosis (Table 1) (18).

**Table 1:** Baseline demographics and clinical variables of study subjects.

Variables	Donepezil (*n* = 60)	Donepezil + Ginkgo (*n* = 41)	*p*-value^*^
Age, years	70.8 ± 9.9	71.9 ± 8.8	0.579
Female gender (%)	32 (53.3%)	22 (53.7%)	0.568
Education years	10.4 ± 5.4	10.4 ± 5.8	0.992
ApoE4 gene number	0.38 ± 0.62	0.39 ± 0.63	0.956
Donepezil dose (mg)	9.6 ± 1.3	9.5 ± 1.5	0.577
K-MMSE	22.7 ± 4.3	21.2 ± 4.9	0.196
CDR	0.7 ± 0.3	0.7 ± 0.3	0.995
CDR-SB	3.4 ± 2.5	3.4 ± 2.6	0.713
MDS-Oaβ (ng/ml)	0.88 ± 0.17	0.87 ± 0.16	0.499

*Between-group comparisons were conducted using independent *t*-tests.

K-MMSE; Korean Mini-Mental State Examination, CDR; Clinical Dementia Rating Scale, CDR-SB; Clinical Dementia Rating Scale Sum of Box, MDS-Oaβ; Multimer Detection System-Oligomerized Aβ assay.

### 3.2 Follow-up clinical outcome after 12 months of medication in study groups

**Figure 2:**
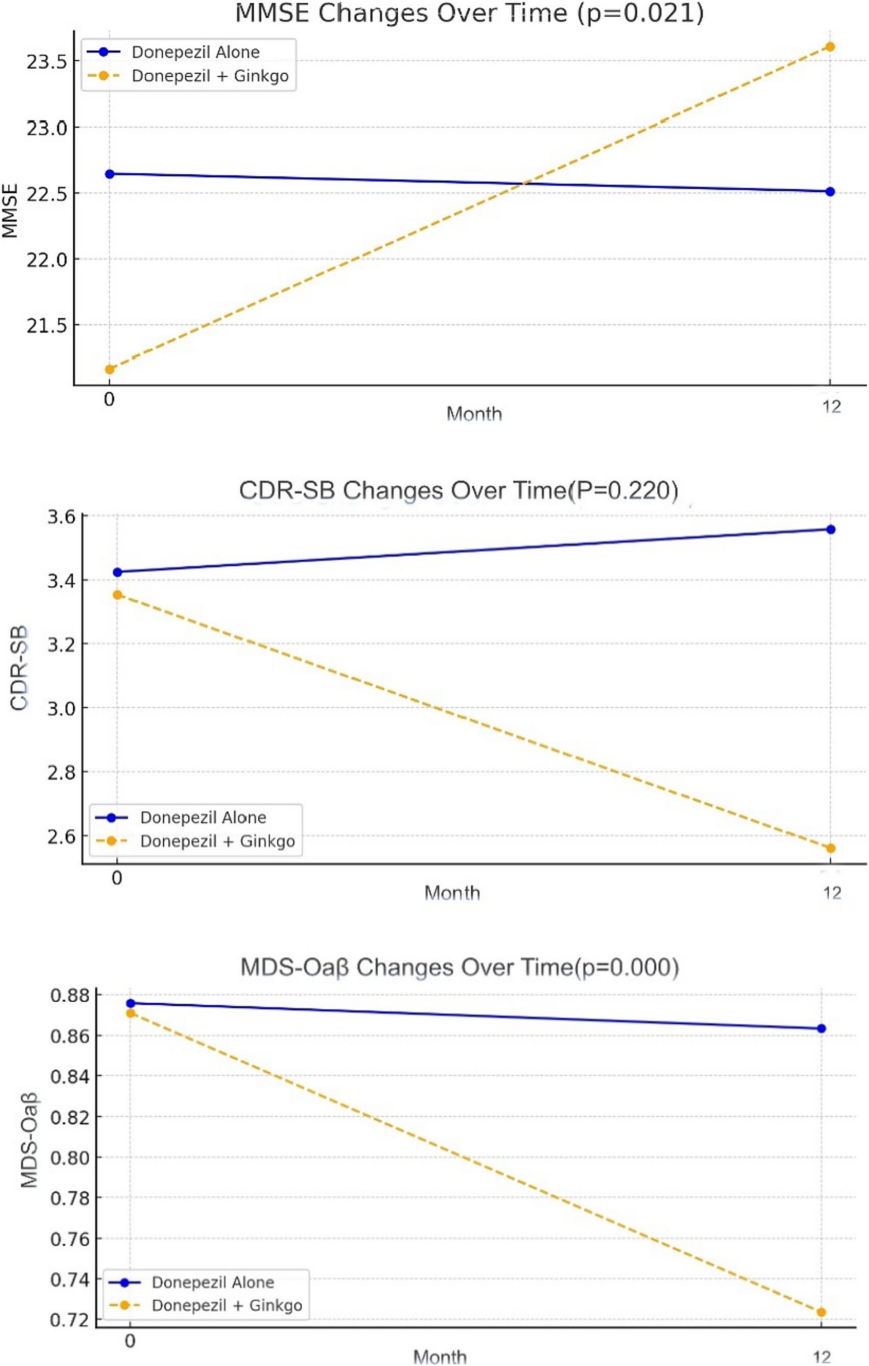
Change of K-MMSE, CDR-SB and MDS-Oaβ in Alzheimer disease patients with donepezil alone and donepezil plus Ginkgo treatment.

After 12 months, 56 patients (93.3%) in the Donepezil-only group and 37 (90.2%) in the Donepezil-Ginkgo group were on a 10 mg/day dose of donepezil; the remainder stayed at 5 mg/day. At 12 months, the Donepezil-only group had K-MMSE = 22.5, CDR-SB = 3.6, MDS-Oaβ = 0.86, indicating changes of −0.1, +0.1, and −0.01 from baseline, respectively. Among these, only the MDS-Oaβ change was statistically significant (*p* = 0.023). In contrast, the Donepezil-Ginkgo group showed K-MMSE = 23.6, CDR-SB = 2.6, and MDS-Oaβ = 0.72, reflecting changes of +2.4, −0.8, and −0.15 from baseline. The improvements in K-MMSE and the reduction in MDS-Oaβ were significant, whereas the change in CDR-SB was not. Moreover, when comparing the two groups at 12 months, K-MMSE (*p* = 0.021) and MDS-Oaβ (*p* < 0.001) showed significant between-group differences (Figure 2 and Table 2).

**Table 2:** Comparison of clinical outcome and MDS-Oaβ between Donepezil alone and Donepezil plus Ginkgo groups over 12 months.

	Group	0 month	12 month	delta	P1^*^	P2^*^
K-MMSE	Donepezil	22.7 ± 4.3	22.5 ± 4.6	−0.1 ± 5.3	0.846	0.021
	Donepezil + Ginkgo	21.2 ± 4.7	23.6 ± 3.3	2.4 ± 5.6	0.008	
CDR-SB	Donepezil	3.4 ± 2.4	3.6 ± 3.3	0.1 ± 3.9	0.792	0.220
	Donepezil + Ginkgo	3.4 ± 2.6	2.6 ± 2.3	−0.8 ± 3.3	0.142	
MDS-Oaβ (ng/ml)	Donepezil	0.88 ± 2.4	0.86 ± 3.3	−0.01 ± 0.04	0.023	0.000
	Donepezil + Ginkgo	0.87 ± 0.16	0.72 ± 0.14	−0.15 ± 0.12	0.000	

*Within-group comparisons (P1) were conducted using paired *t*-tests, and between-group comparisons (P2) were conducted using independent *t*-tests.

P1; Baseline vs. Follow-up *p*-value (within-group).

P2; Donepezil monotherapy vs. Donepezil + Ginkgo combination *p*-value (between-group).

K-MMSE, Korean Mini-Mental State Examination; CDR, Clinical Dementia Rating Scale; CDR-SB, Clinical Dementia Rating Scale Sum of Box; MDS-Oaβ, Multimer Detection System-Oligomerized Aβ assay.

### 3.3 Adverse events after 12 months of study groups

In the Donepezil-only group, 10 patients experienced adverse events, while 9 patients in the Donepezil-Ginkgo group reported adverse events, most of which were gastrointestinal symptoms. In the Donepezil-alone group, dizziness was observed in 3 patients, whereas it was not reported in the Donepezil-Ginkgo group. However, none of these events led to treatment discontinuation (Table 3).

**Table 3:** Adverse events in follow up study group.

Adverse event	Donepezil (*N* = 60)	Donepezil + Ginkgo (*n* = 41)
Diarrhea	4 (6.0%)	5 (12.2%)
Headache	2 (3.0%)	3 (7.3%)
Nausea	2 (3.0%)	4 (9.8%)
Vomiting	1 (1.5%)	1 (2.4%)
Dizziness	3 (4.5%)	0 (0.0%)

In the Donepezil group, 2 patients (3.3%) had multiple adverse events, and in the Donepezil + Ginkgo group, 3 patients (7.3%) had multiple adverse events.

## 4 Discussion

Given the persistent challenges in developing novel treatments for AD, it has become increasingly important to revisit established agents with potential anti-AD properties. Ginkgo is noteworthy for its reported antioxidative, anti-inflammatory, and anti-amyloid effects. However, earlier clinical trials yielded mixed outcomes, possibly due to heterogeneous study populations (e.g., participants without confirmed AD pathology) and the slow, insidious progression of AD that can mask subtle therapeutic effects in short-term studies. To address these issues, our study enrolled only amyloid PET-positive AD patients, thereby confirming pathological involvement, and employed both conventional clinical measures (K-MMSE, CDR-SB) and a biomarker known as MDS-Oaβ. MDS-Oaβ quantifies the propensity of plasma amyloid monomers to form oligomers (17) and correlates with cerebrospinal fluid measures and amyloid PET (19), as well as with overall disease progression (20). Unlike biomarkers such as Aβ42, Aβ40, or tau, which detect specific existing substances, this biomarker evaluates the tendency of how effectively monomeric amyloid in plasma forms into oligomeric amyloid. It provides a comprehensive assessment of the various conditions and stages involved in amyloid oligomerization. This makes it particularly suitable for reflecting the effects of drugs like Ginkgo, which exhibit multifaceted pharmacological actions across different stages of disease progression.

Before analyzing the 12-month outcomes, we first compared baseline characteristics between the Donepezil-only and Donepezil-Ginkgo groups. Although the Donepezil-Ginkgo group tended to have a lower baseline K-MMSE score than the Donepezil-only group (21.2 vs. 22.7), this difference was not statistically significant (*p* = 0.196), and the gap was not large enough to warrant additional statistical adjustments (Table 1). After 12 months of medication therapy, the Donepezil-only group showed minimal change in K-MMSE (−0.1), aligning with the known trajectories of cholinesterase inhibitors in mild-to-moderate AD (21). Meanwhile, a small but statistically significant reduction in MDS-Oaβ was observed in this group, potentially reflecting a natural course or partial effect of donepezil.

In contrast, the addition of Ginkgo yielded a larger MDS-Oaβ reduction (−0.15) and a meaningful K-MMSE increase (+2.4). This suggests additive or synergistic benefits when combining Ginkgo with donepezil, resonating with previous studies where EGb 761 plus standard AD medications slowed progression more effectively than monotherapy (22, 23). Özge et al. (22) found that EGb 761 administered alongside standard AD medications slowed disease progression more effectively than monotherapy, though their cohort included both mild cognitive impairment (MCI) and AD patients. García-Alberca et al. (23) similarly reported cognitive and neuropsychiatric benefits for amnestic MCI patients treated with EGb 761 plus cholinesterase inhibitors.

Mechanistically, the reduction in MDS-Oaβ may be attributable to Ginkgo’s broad protective actions, including its anti-inflammatory effects (24), inhibition of Aβ1-42 aggregation (25), and attenuation of oxidative stress (26). These effects may be achieved through mechanisms such as scavenging free radicals, enhancing cerebral microperfusion, and inhibiting Aβ fibrillogenesis (27, 28). When combined with the cholinesterase inhibition offered by donepezil, these effects may reduce amyloid oligomer formation and bolster cognitive function. Although CDR-SB showed no significant between-group difference, this likely reflects the scale’s lower sensitivity over a 12-month period in mild-stage AD (mean baseline CDR of 0.7). Additionally, no severe adverse events occurred, suggesting that Ginkgo is generally safe to combine with donepezil.

Our study does have limitations, including its retrospective design and relatively small sample size, which preclude definitive causal conclusions and highlight the need for larger, prospective trials. While a Ginkgo-alone group could have provided additional insights, we did not include this group due to ethical concerns regarding withholding standard-of-care treatment for Alzheimer’s disease. However, our study contributes to the growing body of indirect evidence supporting Ginkgo’s potential role in AD treatment. Based on these findings, we plan to design future prospective studies that explore Ginkgo’s effects within ethically feasible frameworks, such as in earlier disease stages or as an adjunct to other treatments. Furthermore, using K-MMSE alone may not fully capture complex cognitive or neuropsychiatric changes, and other confounders (e.g., comorbidities, adherence, lifestyle) could influence outcomes.

## 5 Conclusion

In this retrospective study, our data suggest that combining Ginkgo with donepezil in amyloid PET-positive AD patients may confer tangible cognitive benefits and a more robust decrease in MDS-Oaβ, possibly via reduced amyloid oligomerization tendency. Large-scale, prospective clinical trials with multiple biomarkers, such as tau imaging, inflammation markers, or advanced neuropsychological assessments, are warranted to further validate and clarify Ginkgo’s mechanistic role in AD.

## Data Availability

The raw data supporting the conclusions of this article will be made available by the authors, without undue reservation.
